# Distance simulation in the health professions: a scoping review

**DOI:** 10.1186/s41077-023-00266-z

**Published:** 2023-11-17

**Authors:** Rachel Elkin, Jonathan P. Duff, Marian L. LaForest, Stephanie Stapleton, Geethanjali Ramachandra, Janice C. Palaganas, Isabel T. Gross

**Affiliations:** 1 Division of Pediatric Emergency Medicine, Department of Emergency Medicine, Columbia University Vagelos College of Physicians and Surgeons, New York, NY USA; 2 Division of Pediatric Critical Care Medicine, Department of Pediatrics, University of Alberta, Edmonton, Canada; 3 Augustus Long Health Sciences Library, Columbia University Irving Medical Center, New York, NY USA; 4 Department of Emergency Medicine, Boston University, Boston, MA USA; 5 Department of Pediatric Intensive Care, Krishna Institute of Medical Science, Secunderabad, India; 6 Department of Health Professions Education, MGH Institute of Health Professions, Boston, MA USA; 7 Section of Pediatric Emergency Medicine, Department of Pediatrics, Yale University School of Medicine, New Haven, CT USA

**Keywords:** Distance simulation, Scoping review, Risk of bias

## Abstract

**Background:**

Distance simulation is defined as simulation experiences in which participants and/or facilitators are separated from each other by geographic distance and/or time. The use of distance simulation as an education technique expanded rapidly with the recent COVID-19 pandemic, with a concomitant increase in scholarly work.

**Methods:**

A scoping review was performed to review and characterize the distance simulation literature. With the assistance of an informationist, the literature was systematically searched. Each abstract was reviewed by two researchers and disagreements were addressed by consensus. Risk of bias of the included studies was evaluated using the Risk of Bias 2 (RoB 2) and Risk of Bias in Non-randomized Studies of Interventions (ROBINS-I) tools.

**Results:**

Six thousand nine hundred sixty-nine abstracts were screened, ultimately leading to 124 papers in the final dataset for extraction. A variety of simulation modalities, contexts, and distance simulation technologies were identified, with activities covering a range of content areas. Only 72 papers presented outcomes and sufficient detail to be analyzed for risk of bias. Most studies had moderate to high risk of bias, most commonly related to confounding factors, intervention classification, or measurement of outcomes.

**Conclusions:**

Most of the papers reviewed during the more than 20-year time period captured in this study presented early work or low-level outcomes. More standardization around reporting is needed to facilitate a clear and shared understanding of future distance simulation research. As the broader simulation community gains more experience with distance simulation, more studies are needed to inform when and how it should be used.

**Supplementary Information:**

The online version contains supplementary material available at 10.1186/s41077-023-00266-z.

## Background

Distance simulation—or simulations in which participants are separated from one another by geographic distance and/or time—has been in existence for decades [1, 2]. It allows educators to offer simulation-based education to participants without the barrier of having to gather in the same space. In recent years, adoption of this approach to simulation has accelerated [3–6]. This rapid increase in growth was brought to a head by the COVID-19 pandemic when physical distancing restrictions prompted rapid uptake of distance simulation activities by simulationists across the globe [7].

As distance simulation becomes more widespread and evolves as a scholarly discipline [8], it is important to understand the state of the literature so that gaps, and therefore, future research directions, can be identified. While previous reviews have examined distance learning paradigms [9, 10] and specific simulation modalities (such as virtual reality; [11, 12]), to our knowledge, no review has focused explicitly on the distance simulation literature as a whole.

Our objective with this scoping review is to map out the current state of the distance simulation literature and explore how distance simulation is being utilized in health professions education.

## Methods

Given our stated objectives to map the current state of a rapidly evolving body of literature and to identify gaps/opportunities for future research within this area, we opted to begin with a scoping review [13]. A scoping review approach enables a systematic search of both the peer-reviewed and grey literature, while also effectively accommodating a diverse array of source materials with heterogeneous methods of reporting. The methods of this scoping review followed the five-stage framework laid out by Arksey and O’Malley [14] and refined by Levac and colleagues [15].

### Study team

Our reviewer/author group includes active simulationists and simulation researchers from different countries (USA, Canada, India), clinical backgrounds (medicine, nursing), and specialties (both pediatric- and adult-focused). The reviewers have facilitated simulation and simulation research activities and/or taught about simulation locally, regionally, nationally, and internationally. Several of us also play an active role in planning and organizing an annual distance simulation research summit—now in its fourth year—that convenes a diverse array of experts with a focus on the generation and dissemination of distance simulation scholarship. The reviewers were mixed in different combinations/pairs throughout the stages of the review to maximize the diversity of interactions and perspectives but met regularly as a group to discuss issues that arose to ensure a consistent throughline to the review.

### Identify the research question

This scoping review was conducted to answer the following questions:

- What are the characteristics of distance simulation activities conducted both before and during the pandemic?
- Which types of health professions learners are utilizing distance simulation, and to cover what content?
- What outcomes, if any, do distance simulation researchers measure and report?

### Identify relevant studies

With the assistance of an experienced informationist (MLL), comprehensive database searches (Additional file 1) were run in Cochrane Library, EBSCO, Embase, ERIC (Education Resources Information Center), PubMed, and Scopus in July and September 2020. A grey literature search was also performed in August and September 2020 to identify literature not indexed in traditional databases. The following sources were searched by hand, keywords, and/or hashtags: International Nursing Association for Clinical Simulation and Learning (INACSL), International Pediatric Simulation Society (IPSS), SimGHOSTS, Simulation Canada, Society for Simulation in Europe (SESAM), Society for Simulation in Healthcare (SSH) website, COVID-19 WhatsApp group & Simulation Online 2020 Facebook group (groups created to share simulation resources during the pandemic), and Twitter. Additional articles were identified through hand-searching the reference lists of review articles found to include studies on distance simulation as well as articles identified to be of interest by the study authors during the screening process and through discussions with researchers in the field. No date restrictions were applied except for Twitter, which was only searched back to February 15, 2020. No language restrictions were applied during the search.

### Study selection

Studies that described a distance simulation activity for health professions participants were considered eligible for inclusion. Given the extremely vast yield generated by initial searches, to balance breadth and comprehensiveness with feasibility [15], we restricted the scope of this review to *synchronous* distance simulation activities in which not all participants shared the same physical space. Simulation activities that were entirely asynchronous were excluded. Similarly, studies that described simulations that lacked a distance component (i.e., were exclusively in-person/face-to-face), focused on learning activities outside of simulation or included learners outside of healthcare were excluded from this review.

Resources were screened within Covidence (Veritas Health Innovation, Melbourne, Australia), an online knowledge synthesis tool. Two researchers independently screened each title and abstract for potential inclusion. Similarly, two researchers independently screened each full-text article for potential inclusion. A third researcher was available to help mediate a decision if the two reviewers were unable to reach a decision on abstract or full-text screening.

### Charting the data

A custom template was created within Covidence for data extraction. A small number of full-text articles first were reviewed and the extraction template iterated as needed until consensus was achieved on the nature and extent of data to be extracted. Variables extracted included those related to study demographics (e.g., year of publication, country/countries and professions/disciplines of study authors), study and simulation characteristics (e.g., study design, simulation modality [16], content covered, learner characteristics [profession and specialty/discipline], simulator type [16]), technological considerations (e.g., which elements of the simulation activity included a distance element, how the distance element was achieved, participant configuration), outcomes reported, debriefing method, and assessment characteristics (e.g., target of assessment [simulation and/or learner], assessment tool utilized). Outcomes were classified as reactions (referred to in previous reviews as “attitudes”), knowledge, or skills, as outlined in previous simulation-based education reviews [17–21]. Outcomes assessed within the context of the simulation included time skills (how long it takes a learner to complete a task), process skills (e.g., global rating scales or minor errors), and product skills (e.g., successful completion of a task, quality of the finished product, or major errors). In parallel, outcomes assessed in the clinical space included behavior [time] (time to task completion in the clinical space), behavior [process] (e.g., provider performance rating with patients), and results or direct effects on patients [products] (e.g., procedural completion or procedural errors). For grey literature studies, the type of media represented by the study (e.g., presentation/webinar, podcast, blog) also was collected. A full list of variables extracted can be found in Supplemental Table S1.

After reviewing the first several full-text studies, a number of potential concerns regarding methodological rigor in these studies were identified. Therefore, to provide a more consistent and objective basis for evaluation and comparison of the methodologic rigor of the included studies, risk of bias assessments were completed for studies that reported outcome data. We utilized the Risk of Bias 2 (RoB 2) and Risk of Bias in Non-randomized Studies—of Interventions (ROBINS-I) tools for randomized parallel-group trials, and non-randomized studies, respectively. These tools were selected for their ability to provide both a summary assessment of the overall risk of bias, as well as more granular data regarding which aspects of the studies were contributing to this assessment.

Two researchers independently extracted data and completed risk of bias assessments (when applicable) for each study. Disagreements were resolved by consensus.

### Summarizing and reporting results

A table summarizing the results for each individual article can be found in Supplemental Table S1. Descriptive statistics were calculated to facilitate reporting of patterns and trends in study demographics, study characteristics, study outcomes, debriefing, assessment, and risk of bias. Outcome categories were grouped within a Kirkpatrick framework [22], with reactions classed as level 1; knowledge, time skills, process skills, and product skills as learning (level 2); behavior (time) and behavior (process) as level 3; and results (or direct effects on patients [products]) as level 4. Outcome, debriefing, and assessment data are reported both cumulatively and by the learner profession.

## Results

### Study flow and demographics

**Fig. 1:**
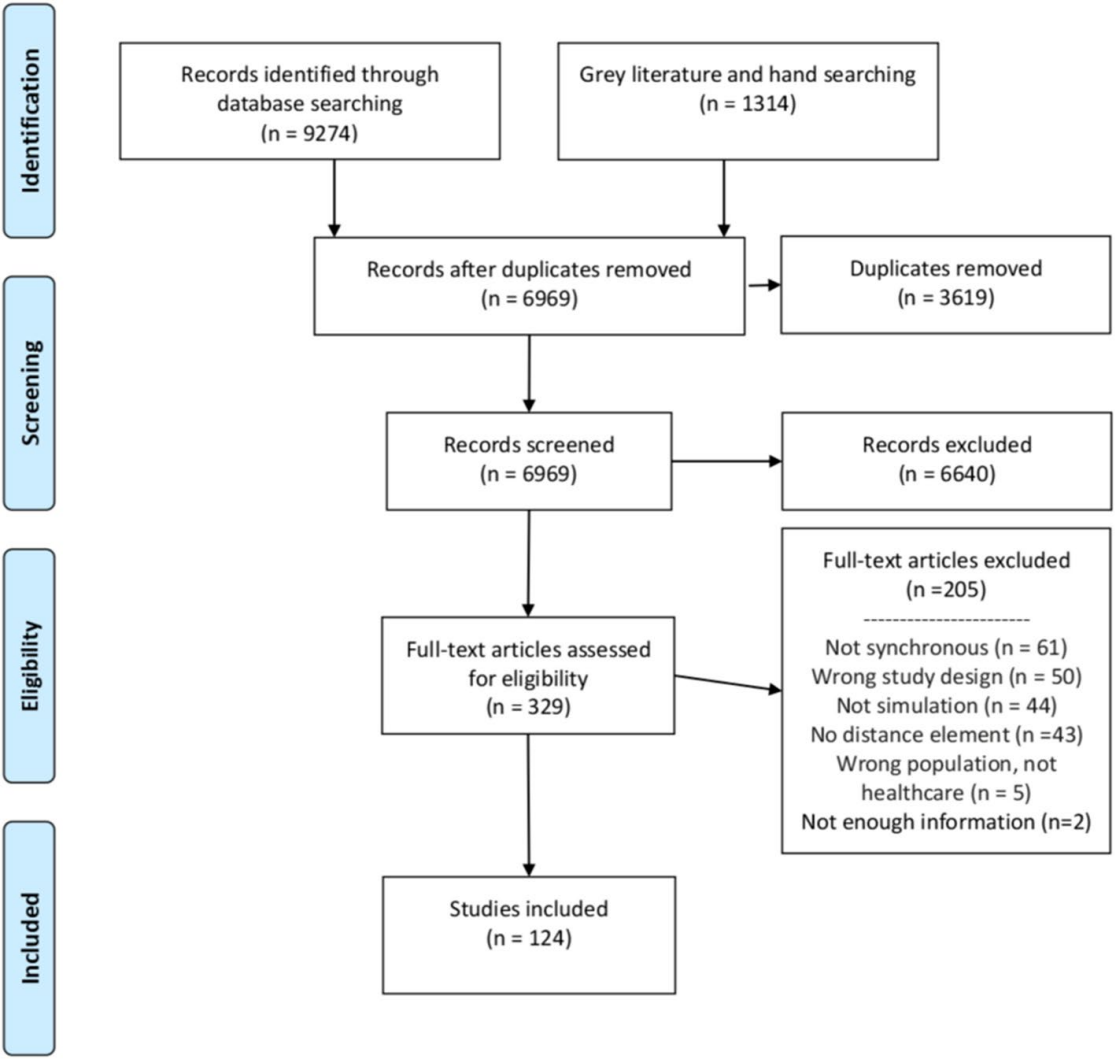
Study flow diagram

Our initial search yielded a total of 6969 unique articles and media resources (“articles”), of which 124 ultimately met the criteria for inclusion (Fig. 1; Supplemental Table S2).

Of the 124 articles included in the dataset for analysis, 64.5% (*n* = 80) were found in the peer-reviewed search and 35.5% (*n* = 44) were found in the grey literature search. While we did not limit our search to the English language, 99% (*n* = 123) of the articles were in English; one article was in French. A number of different terms were utilized by authors to describe distance simulation activities. In order of frequency, the most common terms employed were virtual, distance, remote, and tele-.

**Fig. 2:**
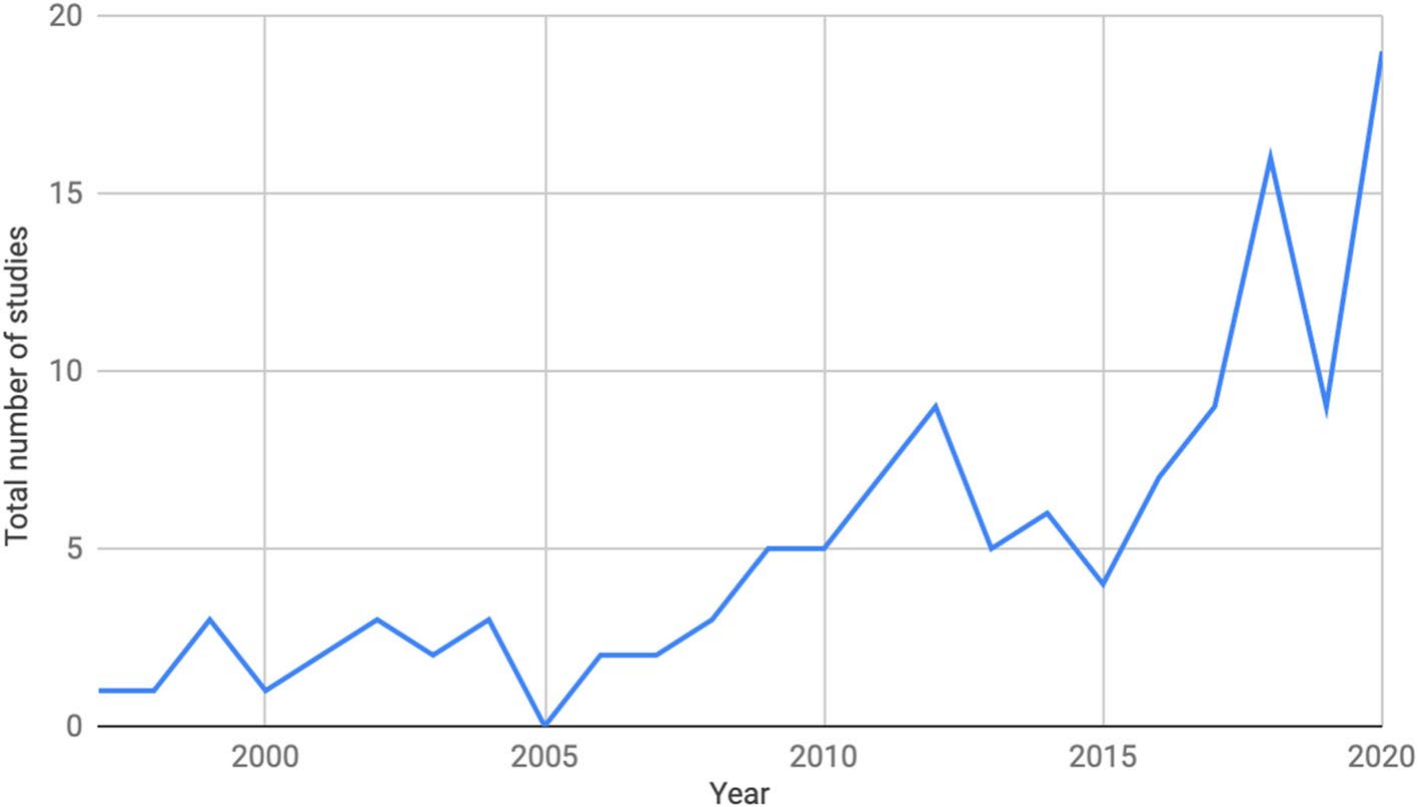
Studies included in the scoping review charted by publication year. A larger upswing of publications occurred between 2015 and 2020, with 2018 and 2020 having the highest number of publications per year

Publication years ranged from 1997 to 2020 (see Fig. 2), with the highest number of studies published in 2020 (*n* = 19) and 2018 (*n* = 16). Most of the articles identified (77%, *n* = 96) were published in North America (61% in the USA, *n* = 76; 16% in Canada, *n* = 20). Of the articles that reported the professional background of their authors, 55% were uniprofessional studies and 45% were interprofessional. The most frequent professions identified by authors included physicians (*n* = 56), registered nurses (*n* = 33), nurse practitioners (*n* = 9), PhD researchers (*n* = 12), education (*n* = 9), and simulation (*n* = 9).

### Study characteristics

Study characteristics are summarized in Table 1. The simulation was the subject of the research in 82% of studies (102/124), the investigational method in 10% (12/124), and was utilized for both purposes in another 10% (12/124). A variety of simulation modalities [16] were employed, including computer-based simulation (27%; 34/124), simulated patient (24%; 30/124), simulated clinical immersion (24%; 30/124), and procedural simulation (21%; 26/124). The remaining four studies used other modalities or did not report the modality (3%; 4/124).

**Table 1:** Characteristics of included studies (*n* = 124)

**Simulation content**	
Acute care/team training	36.3% (45 studies)
Procedural/surgical skills training	28.2% (35 studies)
Outpatient medicine/community training	17.7% (22 studies)
Communication/history taking	8.1% (10 studies)
Disaster preparedness	4.8% (6 studies)
Not reported	4.8% (6 studies)
**Simulator types**	
Patient simulators	25.8% (32 studies)
Technology-enhanced (e.g., extended reality, virtual worlds)	25.8% (32 studies)
Patient actors	20.2% (25 studies)
Task trainers	18.5% (23 studies)
Computer/web applications	8.1% (10 studies)
Not reported	1.6% (2 studies)
**Study design**	
Observational	52.4% (65 studies)
Randomized^a^	10.5% (13 studies)
Non-randomized experimental	20.2% (25 studies)
Concept papers	5.6% (7 studies)
Case studies	5.6% (7 studies)
Qualitative	4.0% (5 studies)
Technical reports	1.6% (2 studies)
**Technology used**	
Web-conferencing (Skype, Zoom, etc.)	38.7% (48 studies)
Telehealth technology	29.8% (37 studies)
Virtual worlds (Second Life, etc.)	15.3% (19 studies)
Custom systems	12.9% (16 studies)
Not adequately described	3.3% (4 studies)

^a^Includes randomized controlled designs that encompass both parallel and crossover designs

The configuration of simulation participants (learners, facilitators, simulated patient/standardized patient (SP), simulation technician/operator, etc.) during distance simulation activities varied widely in the studies reviewed. Twenty-three percent (28/124) of studies were entirely distanced, meaning all participants joined the simulation from a distinct geographic location. Many studies (48%; 60/124) employed a hybrid approach, in which two or more participants joined from a shared geographic location, while others joined from one or more distinct locations. In 24% (29/124) of studies, while certain participants (such as a facilitator or SP) were geographically separated from the learners, it was not clear whether some learners shared the same location or if each individual learner joined from geographically distinct locations. Six percent (7/124) of studies did not provide sufficient information to ascertain the configuration of their participants.

### Outcomes

Table 2 summarizes the outcomes, as well as the debriefing and assessment data, reported in the included studies. Overall, outcomes were reported in 64% (79/124) of studies reviewed. Across learner professions, 68% (15/22) of studies with an interprofessional learner group, 70% (14/20) of studies with nursing learners, 62% (8/13) of studies with nurse practitioner learners, and 66% (31/47) of studies with physician learners reported outcomes (Table 2). Outcomes were broken down further in accordance with the framework described earlier, and are presented grouped by level within the Kirkpatrick model in Table 3.

**Table 2:** Outcome, debrief, and assessment data, were stratified by the professional group of learners

		Learners by profession											
		IP		N/A		Nurse		Nurse practitioner		Other		Physician	
		Count	(%)	Count	(%)	Count	(%)	Count	(%)	Count	(%)	Count	(%)
Outcomes	No outcomes	7	32%	7	100%	6	30%	5	38%	4	27%	16	34%
	Outcomes	15	68%	0	0%	14	70%	8	62%	11	73%	31	66%
	Total	22	1	7	1	20	1	13	1	15	1	47	1
Debrief	Debrief unclear	0	0%	1	14%	0	0%	0	0%	0	0%	1	2%
	No debrief	10	45%	6	86%	4	20%	3	23%	7	47%	23	49%
	Debrief held	12	55%	0	0%	16	80%	10	77%	8	53%	23	49%
	Distance debrief^a^	12	100%	0	0%	12	75%	8	80%	6	75%	18	78%
Assess	Assessment unclear	0	0%	1	14%	0	0%	0	0%	0	0%	0	0%
	No assessment	8	36%	6	86%	7	35%	4	31%	4	27%	13	28%
	Assessment done	14	64%	0	0%	13	65%	9	69%	11	73%	34	72%
	Distance assessment^a^	9	64%	0	0%	9	69%	7	78%	8	73%	23	68%

*IP* Interprofessional learner group, *N/A* learner professional group not specified

^a^As not all studies performed or reported a debrief or assessment, the percentages for the questions marked with an asterisk are calculated as a count per question over the number of studies that reported a debrief or assessment

**Table 3:** Outcome data as organized within a Kirkpatrick framework, stratified by the professional group of learners

			Learners by profession											
			IP		N/A		Nurse		Nurse practitioner		Other		Physician	
	Kirkpatrick level and measures		Count	(%)	Count	(%)	Count	(%)	Count	(%)	Count	(%)	Count	(%)
Outcomes type	I	Reactions	12	80%	0	0%	9	64%	5	63%	9	82%	23	74%
	II	Learning: Knowledge	2	13%	0	0%	2	14%	2	25%	5	45%	6	19%
	II	Learning: Skills (time)	4	27%	0	0%	0	0%	0	0%	1	9%	9	29%
	II	Learning: Skills (process)	5	33%	0	0%	4	29%	3	38%	2	18%	17	55%
	II	Learning: Skills (product)	6	40%	0	0%	1	7%	0	0%	1	9%	7	23%
	III	Behavior (time)	0	0%	0	0%	0	0%	0	0%	0	0%	0	0%
	III	Behavior (process)	1	7%	0	0%	0	0%	0	0%	0	0%	1	3%
	IV	Results: Direct effects on patients (products)	0	0%	0	0%	0	0%	0	0%	0	0%	0	0%

*IP* Interprofessional learner group, *N/A* learner professional group not specified

Percentages are calculated as a count per question over the number of studies that reported an outcome per profession

Reactions were the most commonly included outcome in all learner categories and were described in 73% (58/79) of the identified studies reporting outcomes, whereas knowledge was reported in 22% (17/79) of all studies reporting outcomes. Of the included studies that reported outcomes, 18% (14/79) reported time skills, 39% (31/79) process skills, and 19% (15/79) product skills. In all learner groups, process was the most commonly reported skill category except for interprofessional learners where product skills (40%; 6/15) were more frequently described.

Behavior outcomes were rarely reported and were identified in only 2% (2/17) of all studies with outcome data. Of the two studies that reported behavioral outcomes, one was an interprofessional learner simulation study and the other a physician learner study. None of the included studies reported results-oriented outcomes (i.e., direct effects on patients.)

### Debriefing

Debriefing practices varied across studies: 55% (69/124) studies reported a debrief, 43% (53/124) did not hold a debrief, and in 2% (2/124) studies it was unclear whether or not a debrief was held. Of the 69 studies reporting a debrief, 56 (81%) reported a distance element to the debrief (Table 2). Debriefings were most commonly reported in studies including nursing learners (80%; 16/20) while only 49% (23/57) of physician learner studies reported debriefings. All interprofessional learner studies reported a distance element to the debrief (100%; 12/12), as did most (75%; 12/16) studies with nursing learners.

### Assessment

Sixty-five percent (81/124) of all included studies reported an assessment element and in 69% (56/81) the assessment was performed at a distance (Table 2). Assessments were least common in the interprofessional learner group (with 36% (8/22) not reporting an assessment) and most common in the physician learner group (72%; 34/47). More than half of studies across all learner groups completed their assessments at a distance, with this practice being most frequent with nurse practitioner learners (78%; 7/9).

### Risk of bias assessments

**Fig. 3:**
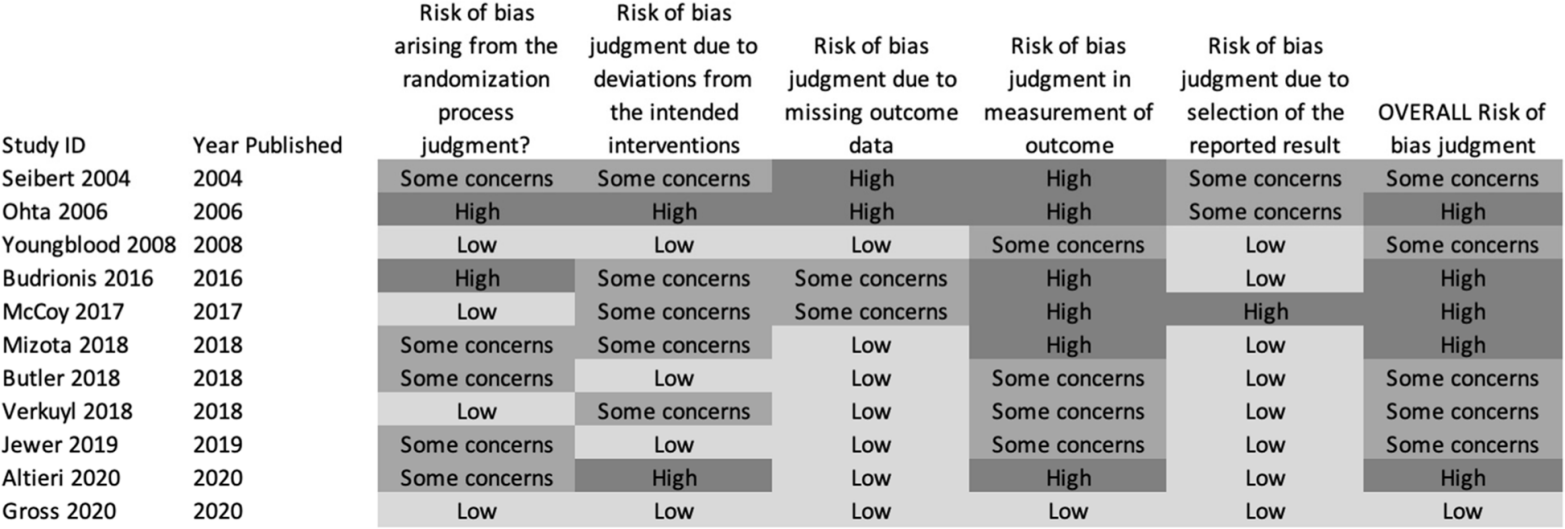
Risk of bias assessments of included randomized trials

**Fig. 4:**
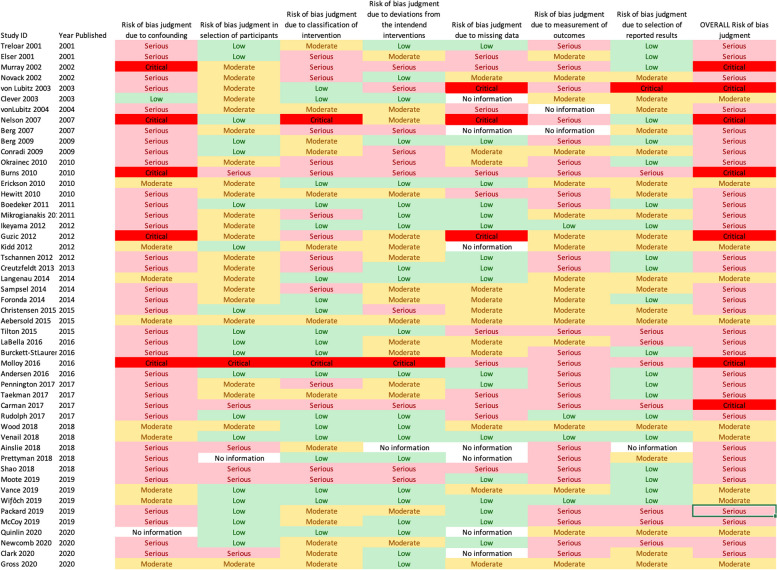
Risk of bias assessments of included non-randomized trials

Of the 124 studies reviewed, 72 studies (58.1%) reported outcomes and had enough information to assess for risk of bias. Twelve studies were randomized trials and were evaluated using the Cochrane ROB-2 tool. Only 1 (1/12; 8.3%) of these 12 studies were found to have an overall low risk of bias. Of the randomized studies that did not meet low-risk criteria, most of this risk of bias was due to issues in randomization or measurement of outcomes (Fig. 3). The remaining 50 studies were non-randomized and were analyzed using the Cochrane ROBINS-I tool. No trials were found to have an overall low risk of bias; most were moderate or serious with seven (7/50; 14.0%) found to have a critical risk of bias. Most of this risk of bias was related to confounding factors, classification of intervention, or measurement of outcomes (Fig. 4).

## Discussion

The COVID-19 pandemic profoundly changed the medical education and simulation landscapes, requiring rapid adaptations to distance learning environments [8]. Evidence regarding best practices for distance simulation activities in particular at the time was unclear and no structured literature review had been performed to our knowledge prior to the pandemic. From an approach initially only used at select centers as early as the late 1990s, distance simulation has evolved as a modality that in recent years has enjoyed wide-ranging and varied applications, both by preference (in the years leading up to the pandemic) and by necessity (with the restrictions put in place in the era of COVID-19). Furthermore, in a recent survey of simulation organizations and programs across the globe, 82% of respondents indicated plans to continue utilizing distance simulation as restrictions relax as part of hybrid offerings [23].

However, while uptake and utilization of distance simulation have increased, and the dissemination of distance simulation work has accelerated—particularly over the last few years—this review suggests that the field remains at an early stage from both methodologic and quality perspectives.

With respect to study design, a large percentage of studies reviewed were best classified as descriptive, feasibility, or proof-of-concept studies, and more than one-third (36%) of studies reviewed did not report any outcomes. When outcomes were reported, most fell within levels 1 and 2 of Kirkpatrick’s pyramid [22], focusing on learner reactions or on knowledge or skills acquired within the context of the simulation. Very few studies included level 3 outcomes (behavioral outcomes translated outside of the simulation context), and none looked at level 4 results-oriented outcomes (direct effects on patients). Moreover, this focus on exploratory study designs and early-stage outcomes did not appear to change significantly over the more than 20 years worth of literature reviewed. To begin maturing distance simulation as a discipline, we encourage the simulation research community to build on this early foundation with more rigorous study designs that not only measure learner satisfaction/reactions or the knowledge and skills acquired during the simulation itself, but also consider the educational impact of distance simulation activities on retention of learning, behavior within the clinical space, and on patient care outcomes.

Quality assessment of studies that reported outcomes revealed significant concerns regarding their risk of bias. Of the 72 studies eligible for quality assessment, only one was found to be at low risk of bias, with most falling into the moderate or severe categories. A small but not insignificant number of studies were found to have a critical risk of bias. Most of this risk of bias was driven by issues with confounding factors, outcome measurement, and classification of interventions. This has implications for the strength and trustworthiness of the evidence generated from this body of work. As distance simulation continues to develop as a discipline, high-quality, methodologically rigorous studies will be important to ensure a solid foundation on which to build, and enhance the confidence in disseminating our findings to the simulation and education communities.

We found that more than 40% of studies did not include or report a debrief. This has significant implications for the educational value of these simulation activities, as debriefing plays a critical part in the learning process during simulation [24]. Given that debriefing and feedback represent key elements to report as part of simulation-based research [25], this also undermines the quality and translatability of the studies themselves. Unexpectedly, studies with exclusively physician learners were less likely to include or report a debrief relative to those with nursing learners or interprofessional learner groups. It is not entirely clear as to why this discrepancy may have occurred, as both physician- and nursing-driven simulation best practice/best evidence publications have highlighted the importance of debriefing [26, 27], though perhaps more visibly by the latter under the aegis of an international nursing-focused simulation organization. However, in the distance setting, a high-quality debrief is perhaps more important than ever, and should be emphasized across all learner groups to promote an optimal simulation experience [28].

Two unexpected findings relate to the professional background of authors and learners and the content focus of the distance simulations reviewed. We were pleased to find that almost half of the studies reviewed were written by interprofessional author teams; interprofessional learner groups were less frequent but still reasonably well-represented. As a field that is still relatively early in its development [8], the representation of diverse perspectives adds richness and helps to ensure that a variety of voices are helping to move the field forward. Secondly, procedural applications were one of the more common areas of focus in the studies reviewed, despite the potential logistical challenges involved when using a distance simulation approach. However, a relatively common indication for the use of distance simulation is to help teach specialized knowledge or skills to learners in areas where the relevant local expertise might not be readily available.

A final challenge we encountered pertains to the heterogeneity of both the terminology utilized to describe distance simulation as well as the ways in which distance simulation studies were reported. We found that a wide variety of terms were used to describe the same phenomenon of distance simulation, which potentially can be confusing and misleading to researchers and readers alike. Similarly, reporting styles were quite varied and frequently missing key details, making it very challenging at times to understand exactly what was done. We propose that publication guidelines considering distance simulation are needed to guide the distance simulation research community when reporting their findings. This could be accomplished by adding extensions to published simulation-based research guidelines. Such guidelines could include a shared or standardized set of terms [29] to utilize when reporting work on distance simulation. To further enhance a shared understanding of the sometimes complex setups needed for distance simulation studies, pictograms can be a valuable aid. In our anecdotal experience, studies using pictograms as part of their methods often were easier to understand. Pictograms can show in a visual illustration what would take many words to explain. Requiring distance simulation publications to include a standardized and culturally universal pictogram clarifying where persons and equipment are located could greatly improve distance simulation-reporting, enhancing comprehensibility and reproducibility.

## Limitations

Our study has some limitations. First, the search strategy for this review did not include articles published after September 2020. Given the rapid uptake in distance simulation efforts since the pandemic, it is quite probable that the simulation landscape has continued to change significantly. Our team is currently performing systematic reviews that build on the work started with this scoping review; these will include studies published from September 2020 onward. We plan to continuously monitor the literature and identify new trends and findings as this important field of simulation-based research further evolves.

Due to the large number of results in our initial search, we opted to limit the scope of this review to synchronous distance simulation studies, and excluded asynchronous studies. Though also part of the larger umbrella of distance simulation, asynchronous simulation, relative to synchronous distance simulation, has greater conceptual differences to most in-person simulation activities and likely represents a distinct body of literature unto itself. Future work will need to examine the asynchronous simulation literature to investigate similarities and differences with synchronous distance simulation.

Even during the time frame searched, it is possible that key innovations in distance simulation were not captured, as those who were performing the work were too busy or otherwise unable to publish their work. We strove to mitigate this by including alternative means of dissemination (social media, podcasts, blogs, etc.) in addition to traditional publication databases as part of our search strategy.

It is also possible that publication bias may limit published findings as simulation educators might not take their work to publication, especially if the finding is that distance simulation is inferior to in-person simulation. However, as a significant number of studies we encountered were primarily descriptive and did not report outcome data, it is not fully clear how relevant this concern is to the distance simulation setting. During our systematic reviews, we are planning to perform an analysis to identify a potential risk of failure to publish negative findings.

Finally, we were unable to perform risk of bias assessments on a significant minority of studies. However, this primarily reflects the fact that most of these studies lacked reportable outcomes.

## Conclusions and future directions

In summary, in our review of the pre-pandemic and early post-pandemic distance simulation literature, we found that while simulationists from a variety of professional backgrounds have undertaken distance simulation work during this more than 20-year time frame, most studies described early-stage work (descriptive or feasibility/proof-of-concept studies), and just over one-third did not report any outcomes. Of those studies that did report outcomes, most focused on lower-level Kirkpatrick outcomes such as reactions and immediate learning. Additionally, a significant minority of studies (more than 40%) did not hold or failed to report a debrief, a key part of the learning process with simulation.

We identified three main areas for future distance simulation research. First, reporting guidelines are needed to help simulationists and researchers adequately communicate distance simulation-based research. Such guidelines should include recommendations with respect to the terminology used to describe distance simulation work to ensure a consistently shared understanding of what is being done. The use of non-textual descriptors such as pictograms may provide further methodologic clarity. Secondly, as in-person simulations continue to return alongside distance offerings, well-designed studies are needed to determine in which contexts distance simulation is most effective. It will be important to collect data on both learning and patient outcomes to understand how distance simulation compares to in-person simulation activities in these regards. Finally, work remains to be done to ascertain how assessment tools and other instruments designed for use with in-person simulations translate to the distance simulation setting. These data, in turn, will help simulation educators (and researchers) by identifying what, if any, adjustments need to be made when planning distance simulation activities as compared to in-person simulation activities in order to promote optimal learning and ultimately, the best possible patient care outcomes.

### Supplementary Information

**Additional file 1.** Database search strategy.

**Additional file 2: Supplemental Table S1.** Summary of extracted data for all studies included in the review. **Supplemental Table S2.** Studies included in the review.

## Data Availability

All data generated or analyzed during this study are included in this published article [and its supplementary information files].

## Abbreviations

RoB 2: Risk of Bias 2 tool
ROBINS-I: Risk of Bias in Non-randomized Studies of Interventions tool
SP: Standardized patient
IP: Interprofessional learner group
INACSL: International Nursing Association for Clinical Simulation and Learning
IPSS: International Pediatric Simulation Society
SESAM: Society for Simulation in Europe
SSH: Society for Simulation in Healthcare
N/A: Not specified
