# Supplementary material for: Distance simulation in the health professions: a scoping review
Source: Adv Simul (Lond). 2023 Nov 17;8:27. doi: 10.1186/s41077-023-00266-z (PMC10656877; doi:10.1186/s41077-023-00266-z)
Supplement: Supplementary file 1 — Additional file 1. Database search strategy. [file 41077_2023_266_MOESM1_ESM.docx]

**Additional File 1**. Database search strategy.

Scopus | Ran on 9.2.20 | Retrieved 1,096 results

**This search needs to be run in the advanced search box or it will malfunction.*

ABS((“online education” OR “online learning” OR “online module*” OR “online course*” OR “online training*” OR “online class*” OR “online instruction*” OR “online universit*” OR “remote education” OR “remote learning” OR “remote module*” OR “remote course*” OR “remote training*” OR “remote class*” OR “remote instruction*” OR “remote universit*” OR “web-based education” OR “web-based learning” OR “web-based module*” OR “web-based course*” OR “web-based training*” OR “web-based class*” OR “web-based instruction*” OR “internet education” OR “internet learning” OR “internet module*” OR “internet course*” OR “internet training*” OR “internet class*” OR “internet instruction*” OR “distance education” OR “distance learning” OR “distance module*” OR “distance course*” OR “distance training*” OR “distance class*” OR “distance instruction*” OR “mobile education” OR “mobile learning” OR “mobile module*” OR “mobile course*” OR “mobile training*” OR “mobile class*” OR “mobile instruction*” OR “virtual education” OR “virtual learning” OR “virtual module*” OR “virtual course*” OR “virtual training*” OR “virtual class*” OR “virtual instruction*” OR “virtual universit*” OR “computer-assisted education” OR “computer-assisted learning” OR “computer-assisted module*” OR “computer-assisted course*” OR “computer-assisted training*” OR “computer-assisted class*” OR “computer-assisted instruction*” OR “tele education” OR “tele learning” OR “tele module*” OR “tele course*” OR “tele training*” OR “tele class*” OR “tele instruction*” OR “teleeducation” OR “telelearning” OR “telemodule*” OR “telecourse*” OR “teletraining*” OR “teleclass*” OR “teleinstruction*” OR “electronic education” OR “electronic learning” OR “electronic module*” OR “electronic course*” OR “electronic training*” OR “electronic class*” OR “electronic instruction*” OR “e education” OR “e learning” OR “e module*” OR “e course*” OR “e training*” OR “e class*” OR “e instruction*” OR “blended learning”) AND (“simulat*” OR “sim” OR “telesimulation” OR “tele simulation” OR “debrief*” OR “mannequin*” OR “manikin*” OR “learner” OR “operator” OR “facilitator”) AND (“healthcare” OR “health”)) OR TITLE((“online education” OR “online learning” OR “online module*” OR “online course*” OR “online training*” OR “online class*” OR “online instruction*” OR “online universit*” OR “remote education” OR “remote learning” OR “remote module*” OR “remote course*” OR “remote training*” OR “remote class*” OR “remote instruction*” OR “remote universit*” OR “web-based education” OR “web-based learning” OR “web-based module*” OR “web-based course*” OR “web-based training*” OR “web-based class*” OR “web-based instruction*” OR “internet education” OR “internet learning” OR “internet module*” OR “internet course*” OR “internet training*” OR “internet class*” OR “internet instruction*” OR “distance education” OR “distance learning” OR “distance module*” OR “distance course*” OR “distance training*” OR “distance class*” OR “distance instruction*” OR “mobile education” OR “mobile learning” OR “mobile module*” OR “mobile course*” OR “mobile training*” OR “mobile class*” OR “mobile instruction*” OR “virtual education” OR “virtual learning” OR “virtual module*” OR “virtual course*” OR “virtual training*” OR “virtual class*” OR “virtual instruction*” OR “virtual universit*” OR “computer-assisted education” OR “computer-assisted learning” OR “computer-assisted module*” OR “computer-assisted course*” OR “computer-assisted training*” OR “computer-assisted class*” OR “computer-assisted instruction*” OR “tele education” OR “tele learning” OR “tele module*” OR “tele course*” OR “tele training*” OR “tele class*” OR “tele instruction*” OR “teleeducation” OR “telelearning” OR “telemodule*” OR “telecourse*” OR “teletraining*” OR “teleclass*” OR “teleinstruction*” OR “electronic education” OR “electronic learning” OR “electronic module*” OR “electronic course*” OR “electronic training*” OR “electronic class*” OR “electronic instruction*” OR “e education” OR “e learning” OR “e module*” OR “e course*” OR “e training*” OR “e class*” OR “e instruction*” OR “blended learning”) AND (“simulat*” OR “sim” OR “telesimulation” OR “tele simulation” OR “debrief*” OR “mannequin*” OR “manikin*” OR “learner” OR “operator” OR “facilitator”) AND (“healthcare” OR “health”))

ERIC | Ran on 9.2.20 | Retrieved 214 results

Searched under title and abstract separately

("online education" OR "online learning" OR "online module*" OR "online course*" OR "online training*" OR "online class*" OR "online instruction*" OR "online universit*" OR "remote education" OR "remote learning" OR "remote module*" OR "remote course*" OR "remote training*" OR "remote class*" OR "remote instruction*" OR "remote universit*" OR "web-based education" OR "web-based learning" OR "web-based module*" OR "web-based course*" OR "web-based training*" OR "web-based class*" OR "web-based instruction*" OR "internet education" OR "internet learning" OR "internet module*" OR "internet course*" OR "internet training*" OR "internet class*" OR "internet instruction*" OR "distance education" OR "distance learning" OR "distance module*" OR "distance course*" OR "distance training*" OR "distance class*" OR "distance instruction*" OR "mobile education" OR "mobile learning" OR "mobile module*" OR "mobile course*" OR "mobile training*" OR "mobile class*" OR "mobile instruction*" OR "virtual education" OR "virtual learning" OR "virtual module*" OR "virtual course*" OR "virtual training*" OR "virtual class*" OR "virtual instruction*" OR "virtual universit*" OR "computer-assisted education" OR "computer-assisted learning" OR "computer-assisted module*" OR "computer-assisted course*" OR "computer-assisted training*" OR "computer-assisted class*" OR "computer-assisted instruction*" OR "tele education" OR "tele learning" OR "tele module*" OR "tele course*" OR "tele training*" OR "tele class*" OR "tele instruction*" OR "teleeducation" OR "telelearning" OR "telemodule*" OR "telecourse*" OR "teletraining*" OR "teleclass*" OR "teleinstruction*" OR "electronic education" OR "electronic learning" OR "electronic module*" OR "electronic course*" OR "electronic training*" OR "electronic class*" OR "electronic instruction*" OR "e education" OR "e learning" OR "e module*" OR "e course*" OR "e training*" OR "e class*" OR "e instruction*" OR "blended learning" OR DE "Blended Learning" OR DE "Distance Education" OR DE "Correspondence Study" OR DE "Computer Assisted Instruction" OR DE "Intelligent Tutoring Systems" OR DE "Telecourses" OR DE "Online Courses" OR DE "Virtual Classrooms" OR DE "Virtual Universities") AND ("simulat*" OR "sim" OR DE "Simulation" OR "telesimulation" OR "tele simulation" OR "debrief*" OR "mannequin*" OR "manikin*" OR "learner" OR "operator" OR "facilitator") AND (“healthcare” OR “health” OR DE "Health" OR DE "Child Health" OR DE "Mental Health" OR DE "Occupational Safety and Health" OR DE "Physical Health" OR DE "Public Health" OR DE "Wellness" OR DE "Health Services" OR DE "Community Health Services" OR DE "Hospices (Terminal Care)" OR DE "Medical Services" OR DE "Prenatal Care" OR DE "School Health Services")

EBSCO | Ran on 9.2.20 | Retrieved 1,364 results

Searched under title and abstract separately. Searched all databases on EBSCO.

("online education" OR "online learning" OR "online module*" OR "online course*" OR "online training*" OR "online class*" OR "online instruction*" OR "online universit*" OR "remote education" OR "remote learning" OR "remote module*" OR "remote course*" OR "remote training*" OR "remote class*" OR "remote instruction*" OR "remote universit*" OR "web-based education" OR "web-based learning" OR "web-based module*" OR "web-based course*" OR "web-based training*" OR "web-based class*" OR "web-based instruction*" OR "internet education" OR "internet learning" OR "internet module*" OR "internet course*" OR "internet training*" OR "internet class*" OR "internet instruction*" OR "distance education" OR "distance learning" OR "distance module*" OR "distance course*" OR "distance training*" OR "distance class*" OR "distance instruction*" OR "mobile education" OR "mobile learning" OR "mobile module*" OR "mobile course*" OR "mobile training*" OR "mobile class*" OR "mobile instruction*" OR "virtual education" OR "virtual learning" OR "virtual module*" OR "virtual course*" OR "virtual training*" OR "virtual class*" OR "virtual instruction*" OR "virtual universit*" OR "computer-assisted education" OR "computer-assisted learning" OR "computer-assisted module*" OR "computer-assisted course*" OR "computer-assisted training*" OR "computer-assisted class*" OR "computer-assisted instruction*" OR "tele education" OR "tele learning" OR "tele module*" OR "tele course*" OR "tele training*" OR "tele class*" OR "tele instruction*" OR "teleeducation" OR "telelearning" OR "telemodule*" OR "telecourse*" OR "teletraining*" OR "teleclass*" OR "teleinstruction*" OR "electronic education" OR "electronic learning" OR "electronic module*" OR "electronic course*" OR "electronic training*" OR "electronic class*" OR "electronic instruction*" OR "e education" OR "e learning" OR "e module*" OR "e course*" OR "e training*" OR "e class*" OR "e instruction*" OR "blended learning") AND ("simulat*" OR "sim" OR "telesimulation" OR "tele simulation" OR "debrief*" OR "mannequin*" OR "manikin*" OR "learner" OR "operator" OR "facilitator") AND (“healthcare” OR “health”)

PubMed | Ran on 7.27.20 | Retrieved 3815 results

("online education"[TIAB] OR "online learning"[TIAB] OR "online module*"[TIAB] OR "online course*"[TIAB] OR "online training*"[TIAB] OR "online class*"[TIAB] OR "online instruction*"[TIAB] OR "online universit*"[TIAB] OR "remote education"[TIAB] OR "remote learning"[TIAB] OR "remote module*"[TIAB] OR "remote course*"[TIAB] OR "remote training*"[TIAB] OR "remote class*"[TIAB] OR "remote instruction*"[TIAB] OR "remote universit*"[TIAB] OR "web-based education"[TIAB] OR "web-based learning"[TIAB] OR "web-based module*"[TIAB] OR "web-based course*"[TIAB] OR "web-based training*"[TIAB] OR "web-based class*"[TIAB] OR "web-based instruction*"[TIAB] OR "internet education"[TIAB] OR "internet learning"[TIAB] OR "internet module*"[TIAB] OR "internet course*"[TIAB] OR "internet training*"[TIAB] OR "internet class*"[TIAB] OR "internet instruction*"[TIAB] OR "distance education"[TIAB] OR "distance learning"[TIAB] OR "distance module*"[TIAB] OR "distance course*"[TIAB] OR "distance training*"[TIAB] OR "distance class*"[TIAB] OR "distance instruction*" OR "mobile education"[TIAB] OR "mobile learning"[TIAB] OR "mobile module*"[TIAB] OR "mobile course*"[TIAB] OR "mobile training*"[TIAB] OR "mobile class*"[TIAB] OR "mobile instruction*"[TIAB] OR "virtual education"[TIAB] OR "virtual learning"[TIAB] OR "virtual module*"[TIAB] OR "virtual course*"[TIAB] OR "virtual training*"[TIAB] OR "virtual class*"[TIAB] OR "virtual instruction*"[TIAB] OR "virtual universit*"[TIAB] OR "computer-assisted education"[TIAB] OR "computer-assisted learning"[TIAB] OR "computer-assisted module*"[TIAB] OR "computer-assisted course*"[TIAB] OR "computer-assisted training*"[TIAB] OR "computer-assisted class*"[TIAB] OR "computer-assisted instruction*"[TIAB] OR "tele education"[TIAB] OR "tele learning"[TIAB] OR "tele module*"[TIAB] OR "tele course*"[TIAB] OR "tele training*"[TIAB] OR "tele class*"[TIAB] OR "tele instruction*"[TIAB] OR "teleeducation"[TIAB] OR "telelearning"[TIAB] OR "telemodule*"[TIAB] OR "telecourse*"[TIAB] OR "teletraining*"[TIAB] OR "teleclass*"[TIAB] OR "teleinstruction*"[TIAB] OR "electronic education"[TIAB] OR "electronic learning"[TIAB] OR "electronic module*"[TIAB] OR "electronic course*"[TIAB] OR "electronic training*"[TIAB] OR "electronic class*"[TIAB] OR "electronic instruction*"[TIAB] OR "e education"[TIAB] OR "e learning"[TIAB] OR "e module*"[TIAB] OR "e course*"[TIAB] OR "e training*"[TIAB] OR "e class*"[TIAB] OR "e instruction*"[TIAB] OR "blended learning"[TIAB] OR "Education, Distance"[Mesh] OR "Computer-Assisted Instruction"[Mesh]) AND ("simulat*"[TIAB] OR "sim"[TIAB] OR "Simulation Training"[Mesh] OR "telesimulation"[TIAB] OR "tele simulation"[TIAB] OR "debrief*"[TIAB] OR "mannequin*"[TIAB] OR "manikin*"[TIAB] OR "Manikins"[Mesh] OR "learner"[TIAB] OR "operator"[TIAB] OR "facilitator"[TIAB])

EMBASE | Ran on 7.27.20 | Retrieved 2456 results

(‘online education':AB,TI OR 'online learning':AB,TI OR 'online module*':AB,TI OR 'online course*':AB,TI OR 'online training*':AB,TI OR 'online class*':AB,TI OR 'online instruction*':AB,TI OR 'online universit*':AB,TI OR 'remote education':AB,TI OR 'remote learning':AB,TI OR 'remote module*':AB,TI OR 'remote course*':AB,TI OR 'remote training*':AB,TI OR 'remote class*':AB,TI OR 'remote instruction*':AB,TI OR 'remote universit*':AB,TI OR 'web-based education':AB,TI OR 'web-based learning':AB,TI OR 'web-based module*':AB,TI OR 'web-based course*':AB,TI OR 'web-based training*':AB,TI OR 'web-based class*':AB,TI OR 'web-based instruction*':AB,TI OR 'internet education':AB,TI OR 'internet learning':AB,TI OR 'internet module*':AB,TI OR 'internet course*':AB,TI OR 'internet training*':AB,TI OR 'internet class*':AB,TI OR 'internet instruction*':AB,TI OR 'distance education':AB,TI OR 'distance learning':AB,TI OR 'distance module*':AB,TI OR 'distance course*':AB,TI OR 'distance training*':AB,TI OR 'distance class*':AB,TI OR 'distance instruction*' OR 'mobile education':AB,TI OR 'mobile learning':AB,TI OR 'mobile module*':AB,TI OR 'mobile course*':AB,TI OR 'mobile training*':AB,TI OR 'mobile class*':AB,TI OR 'mobile instruction*':AB,TI OR 'virtual education':AB,TI OR 'virtual learning':AB,TI OR 'virtual module*':AB,TI OR 'virtual course*':AB,TI OR 'virtual training*':AB,TI OR 'virtual class*':AB,TI OR 'virtual instruction*':AB,TI OR 'virtual universit*':AB,TI OR 'computer-assisted education':AB,TI OR 'computer-assisted learning':AB,TI OR 'computer-assisted module*':AB,TI OR 'computer-assisted course*':AB,TI OR 'computer-assisted training*':AB,TI OR 'computer-assisted class*':AB,TI OR 'computer-assisted instruction*':AB,TI OR 'tele education':AB,TI OR 'tele learning':AB,TI OR 'tele module*':AB,TI OR 'tele course*':AB,TI OR 'tele training*':AB,TI OR 'tele class*':AB,TI OR 'tele instruction*':AB,TI OR 'teleeducation':AB,TI OR 'telelearning':AB,TI OR 'telemodule*':AB,TI OR 'telecourse*':AB,TI OR 'teletraining*':AB,TI OR 'teleclass*':AB,TI OR 'teleinstruction*':AB,TI OR 'electronic education':AB,TI OR 'electronic learning':AB,TI OR 'electronic module*':AB,TI OR 'electronic course*':AB,TI OR 'electronic training*':AB,TI OR 'electronic class*':AB,TI OR 'electronic instruction*':AB,TI OR 'e education':AB,TI OR 'e learning':AB,TI OR 'e module*':AB,TI OR 'e course*':AB,TI OR 'e training*':AB,TI OR 'e class*':AB,TI OR 'e instruction*':AB,TI OR 'blended learning':AB,TI OR 'virtual learning environment'/exp OR 'e-learning'/exp) AND ('simulat*':AB,TI OR 'sim':AB,TI OR 'simulation training'/exp OR 'telesimulation':AB,TI OR 'tele simulation':AB,TI OR 'debrief*':AB,TI OR 'mannequin*':AB,TI OR 'manikin*':AB,TI OR 'Manikin'/exp OR 'learner':AB,TI OR 'operator':AB,TI OR 'facilitator':AB,TI)

Cochrane Library | Ran on 7.27.20 | Retrieved 328 results

1. "online education" OR "online learning" OR "online module*" OR "online course*" OR "online training*" OR "online class*" OR "online instruction*" OR "online universit*" OR "remote education" OR "remote learning" OR "remote module*" OR "remote course*" OR "remote training*" OR "remote class*" OR "remote instruction*" OR "remote universit*" OR "web-based education" OR "web-based learning" OR "web-based module*" OR "web-based course*" OR "web-based training*" OR "web-based class*" OR "web-based instruction*" OR "internet education" OR "internet learning" OR "internet module*" OR "internet course*" OR "internet training*" OR "internet class*" OR "internet instruction*" OR "distance education" OR "distance learning" OR "distance module*" OR "distance course*" OR "distance training*" OR "distance class*" OR "distance instruction*" OR "mobile education" OR "mobile learning" OR "mobile module*" OR "mobile course*" OR "mobile training*" OR "mobile class*" OR "mobile instruction*" OR "virtual education" OR "virtual learning" OR "virtual module*" OR "virtual course*" OR "virtual training*" OR "virtual class*" OR "virtual instruction*" OR "virtual universit*" OR "computer-assisted education" OR "computer-assisted learning" OR "computer-assisted module*" OR "computer-assisted course*" OR "computer-assisted training*" OR "computer-assisted class*" OR "computer-assisted instruction*" OR "tele education" OR "tele learning" OR "tele module*" OR "tele course*" OR "tele training*" OR "tele class*" OR "tele instruction*" OR "teleeducation" OR "telelearning" OR "telemodule*" OR "telecourse*" OR "teletraining*" OR "teleclass*" OR "teleinstruction*" OR "electronic education" OR "electronic learning" OR "electronic module*" OR "electronic course*" OR "electronic training*" OR "electronic class*" OR "electronic instruction*" OR "e education" OR "e learning" OR "e module*" OR "e course*" OR "e training*" OR "e class*" OR "e instruction*" OR "blended learning"
2. "Education, Distance"[Mesh]
3. "Computer-Assisted Instruction"[Mesh]
4. #1 OR #2 OR #3
5. "simulat*" OR "sim" OR "telesimulation" OR "tele simulation" OR "debrief*" OR "mannequin*" OR "manikin*" OR "learner" OR "operator" OR "facilitator"
6. "Manikins"[Mesh]
7. "Simulation Training"[Mesh]
8. #5 OR #6 OR #7

#4 AND #8
