# Supplementary material for: Distance simulation in the health professions: a scoping review
Source: Adv Simul (Lond). 2023 Nov 17;8:27. doi: 10.1186/s41077-023-00266-z (PMC10656877; doi:10.1186/s41077-023-00266-z)
Supplement: Supplementary file 2 — Additional file 2: Supplemental Table S1. Summary of extracted data for all studies included in the review. Supplemental Table S2. Studies included in the review. [file 41077_2023_266_MOESM2_ESM.zip › Supplemental Table 2_ESM.docx]

**Supplemental Table 2**. Studies included in the review.

| **First Author** | **Journal/Source** | **Year** | **Article Title** |
| --- | --- | --- | --- |
| Aebersold | JMIR Serious Games | 2015 | Using a Virtual Environment to Deliver Evidence-BasedInterventions: The Facilitator's Experience |
| Ahmed | Simulation in Healthcare | 2016 | Coaching from the sidelines: examining the impact of teledebriefing in simulation-based training |
| Ainslie | Journal of the American Association of Nurse Practitioners | 2018 | Telemedicine simulation in online family nurse practitioner education: Clinical competency and technology integration |
| Altieri | Surgical Endoscopy | 2020 | Educational value of telementoring for a simulation-based fundamental use of surgical energy (FUSE) curriculum: a randomized controlled trial in surgical trainees |
| Alverson | Simulation in Healthcare | 2008 | Medical Students Learn Over Distance Using Virtual Reality Simulation |
| Andersen | Surgery | 2016 | Medical telementoring using an augmented reality transparent display |
| Andreae | Data in Brief | 2020 | Data and debriefing observations on healthcare simulation to prepare for the COVID-19 pandemic |
| Austin | 3rd Annual Sunnybrook Education Conference: Technology Enhanced Learning | 2014 | 'Telesimulation' in Emergency Medicine: connecting Canadian faculty to Ethiopian residents to provide procedural teaching |
| Autry | Obstetrics and Gynecology | 2013 | Teaching Surgical Skills Using Video Internet Communication in a Resource-Limited Setting |
| Balmaks | BMJ Simul Technol Enhanc Learn | 2020 | Remote rapid cycle deliberate practice simulation training during the COVID-19 pandemic |
| Baylis |  | 2020 | Virtual Resus Room |
| Beissel | Anaesth Crit Care Pain Med | 2017 | A trans-atlantic high-fidelity mannequin based telesimulation experience |
| Berg | Journal of Telemedicine and Telecare | 2007 | Standardized patient interviewing with remote interactive technologies |
| Berg | Studies in Health Technology Informatics | 2009 | Remote Videolaryngoscopy Skills Training for Pre-hospital Personnel |
| Berndt | Nursing Education Perspectives | 2015 | Collaborative Classroom Simulation (CCS): An Innovative Pedagogy Using Simulation in Nursing Education |
| Boedeker | Studies in Health Technology and Infomatics | 2011 | The combined use of Skype and the STORZ CMAC video laryngoscope in field intubation training with the Nebraska National Air Guard |
| Budrionis | Journal of Telemedicine and Telecare | 2016 | Assessing the impact of telestration on surgical telementoring: A randomized controlled trial |
| Bulik | Teaching and Learning in Medicine | 2002 | Clinical Performance Assessment and Interactive Video Teleconferencing: An Iterative Exploration |
| Burckett-St Laurent | Acta Anaesthesiology Scand | 2016 | Teaching ultrasound-guided regional anesthesia remotely: a feasibility study |
| Burns | International Journal of Nursing Education Scholarship | 2010 | Leveraging Differences for Collaborative Advantage: Enhancing Student Learning through an International Educational Collaboration |
| Butler | Telemedicine and e-Health | 2018 | The impact of telemedicine on teamwork and workload in pediatric resuscitation: a simulation-based, randomized controlled study |
| Carman | Dimens Crit Care Nurs | 2017 | Use of a Virtual Learning Platform for Distance-Based Simulation in an Acute Care Nurse Practitioner Curriculum |
| Choy | Surgical Endoscopy | 2013 | Remote evaluation of laparoscopic performance using the global operative assessment of laparoscopic skills |
| Christensen | Simulation in Healthcare | 2018 | Learners' Perceptions During Simulation-Based Training An Interview Study Comparing Remote Versus Locally Facilitated Simulation-Based Training |
| Christensen | Simulation in Healthcare | 2015 | Remotely Versus Locally Facilitated Simulation-based Training in Management of the Deteriorating Patient by Newly Graduated Health Professionals |
| Clark | Comprehensive Healthcare Simulation: Implementing Best Practices in Standardized Patient Methodology | 2020 | Zooming with SPs in COVID-19 Response: Using Zoom to train standardized patients and implement formative Objective Structured Clinical Examination with health science students |
| Clever | Medical Education | 2003 | Evaluating surgeons' informed decision making skills: pilot test using a videoconferenced standardised patient |
| Conradi | Medical Teacher | 2009 | Virtual patients in a virtual world: Training paramedic students for practice |
| Cooper | Journal of Clinical Anesthesia | 2000 | Video Teleconferencing With Realistic Simulation for Medical Education |
| Creutzfeldt | Journal of Medical Internet Research | 2013 | Cardiopulmonary Resuscitation Training in High School Using Avatars in Virtual Worlds: An International Feasibility Study |
| Danesh | J Psychosoc Nurs Ment Health Serv | 2019 | Telehealth in Mental Health Nursing Education: Health Care Simulation With Remote Presence Technology |
| DaSilva | Clinical Simulation in Nursing | 2020 | A Programmatic Approach to the Design of a Video Simulation Case Study |
| de Barbara | Global Telehealth 2012: Delivering Quality Healthcare Anywhere Through Telehealth: Selected Papers from Global Telehealth | 2012 | Virtual Simulation Training Using the Storz C-HUB to Support Distance Airway Training for the Spanish Medical Corps and NATO Partners |
| Donohue | Children (Basel, Switzerland) | 2019 | Use of Telemedicine to Improve Neonatal Resuscitation |
| Elser | Journal of Education in Perioperative Medicine | 2001 | Simulated Crisis in Obstetric Anesthesia: Design and Evaluation of a Distance Education Presentation |
| Erickson | Telemed J E Health | 2010 | A Hybrid Integrated Services Digital Network-Internet Protocol Solution for Resident Education |
| Everett | Simulation in Healthcare | 2013 | Transcontinental Telesimulation: The Global Proliferation of the Managing Emergencies in Pediatric Anesthesia (MEPA Course) |
| Foronda | Nurse Education Today | 2014 | Use of virtual clinical simulation to improve communication skills of baccalaureate nursing students: A pilot study |
| Gambadauro | European J Obstet Gynecol Reprod Biol | 2008 | NEST (network enhanced surgical training): A PC-basedsystem for telementoring in gynaecological surgery |
| Garland | Cureus | 2019 | The Application of Low-fidelity Chest Tube Insertion Using Remote Telesimulation in Training Healthcare Professionals |
| Gordon | Clinical Simulation in Nursing | 2017 | Debriefing Virtual Simulation Using an Online Conferencing Platform: Lessons Learned |
| Gross | BMJ Simul Technol Enhanc Learn | 2020 | Telementoring for remote simulation instructor training and faculty development using telesimulation |
| Gross | NEO | 2020 | Comparison of two telemedicine delivery modes for neonatal resuscitation support: a simulation-based randomized trial |
| Guzic | Clinical Simulation in Nursing | 2012 | Distance Learning and Clinical Simulation in Senior Baccalaureate Nursing Education |
| Haginoya | Frontiers in Psychology | 2020 | Online Simulation Training of Child Sexual Abuse Interviews With Feedback Improves Interview Quality in Japanese University Students |
| Hayden | Academic Emergency Medicine | 2018 | Mannequin-based Telesimulation: Increasing Access to Simulation-based Education |
| Hewitt | Innovate: Journal of Online Education | 2009 | Preparing Graduate Students for Virtual World Simulations: Exploring the Potential of an Emerging Technology |
| Hewitt | [Advanced ICTs for Disaster Management and Threat Detection: Collaborative and Distributed Frameworks](https://www.igi-global.com/book/advanced-icts-disaster-management-threat/40265) | 2010 | Incident and Disaster Management Training: Collaborative Learning Opportunities Using Virtual World Scenarios |
| Hill | Studies in Health Technology and Informatics | 1998 | Telepresence Interface with Applications to Microsurgery and Surgical Simulation |
| Honey | Clinical Simulation in Nursing | 2012 | Teaching with Second Life: Hemorrhage Management as an Example of a Process for Developing Simulations for Multiuser Virtual Environments |
| Huun | Clinical Simulation in Nursing | 2018 | Virtual simulations in online nursing education: align with quality matters |
| Ikeyama | Simulation in Healthcare | 2012 | Low-Cost and Ready-to-Go Remote-Facilitated Simulation-Based Learning |
| Jewer | Journal of Medical Internet Research | 2019 | Evaluation of a Mobile Telesimulation Unit to Train Rural and Remote Practitioners on High-Acuity Low-Occurrence Procedures: Pilot Randomized Controlled Trial |
| Kidd | Journal of Interactive Online Learning | 2012 | Development of a Mental Health Nursing Simulation: Challenges and Solutions |
| Kneebone | Medical Teacher | 2009 | Learner-centred feedback using remote assessment of clinical procedures |
| LaBella | Stroke | 2016 | Using a Tele-Presence Platform as a Neuro-ICU Nursing Education Tool: a Collaboration Between Two Comprehensive Stroke Centers |
| Langenau | Patient Education | 2014 | Web-based objective structured clinical examination with remote standardized patients and Skype: resident experience. |
| LeFlore | Clinical Simulation in Nursing | 2014 | Remote-Controlled Distance Simulation Assessing Neonatal Provider Competence: A Feasibility Testing |
| Lentz | Academic Medicine | 1999 | Using telemedicine and standardized patients to evaluate off-campus students' skills |
| M Simulation | https://z.umn.edu/msflexplan | 2020 | M Simulation: Flexible Operations Plan in the COVID-19 Response |
| Marescaux | Bulletin of the Academy of Natural Medicine | 1999 | The Virtual University applied to telesurgery: from tele-education to tele-manipulation |
| Mason | Podcast | 2020 | The Human Performance Podcast #14 Towards Remote Medical Simulation |
| McCallum | Nurse Education Today | 2011 | Exploring nursing students' decision-making skills whilst in a Second Life clinical simulation laboratory |
| McCoy | Western Journal of Emergency Medicine | 2017 | Prospective Randomized Crossover Study of Telesimulation Versus Standard Simulation for Teaching Medical Students the Management of Critically Ill Patients |
| McCoy | AEM Educ Train | 2019 | Feasibility of Telesimulation and Google Glass for Mass Casualty Triage Education and Training |
| McDonald | Scopus | 2012 | Multidiscipline role-play in a 3D virtual learning environment: Experiences with a large cohort of healthcare students |
| Mikrogianakis | Academic Emergency Medicine | 2011 | Telesimulation: An Innovative and Effective Tool for Teaching Novel Intraosseous Insertion Techniques in Developing Countries |
| Mizota | American Journal of Surgery | 2018 | Step-by-step training in basic laparoscopic skills using two-way web conferencing software for remote coaching: A multicenter randomized controlled study |
| Molloy | Stud Health Technol Inform | 2016 | An Innovative Use of Telepresence Robots for Educating Healthcare Professional |
| Moote | Curr Pharm Teach Learn | 2019 | Interprofessional education telephone simulation for campus-based pharmacy students and distance-learning family nurse practitioner students |
| Murray | Journal of Education in Perioperative Medicine | 2002 | Leadership Training: A New Application of Crisis Resource Management and Distance Education in a Large Group Format at a Medical Simulation Facility |
| Musclow | Pain Research Management | 2012 | Vital (Virtual Interactive teaching and learning) pain assessment |
| Naik | Simulation & Gaming | 2020 | Telesimulation for COVID-19 Ventilator Management Training With Social-Distancing Restrictions During the Coronavirus Pandemic |
| Nelson | International Journal of Nursing Education Scholarship | 2007 | The Power of Online Role-Play Simulations: Technology in Nursing Education |
| Newcomb | Journal of Surgical Education | 2020 | Building Rapport and Earning the Surgical Patient's Trust in the Era of Social Distancing: Teaching Patient-Centered Communication During Video Conference Encounters to Medical Students. |
| None listed (Linden Research, Inc.) | www.secondlifegrid.net.s3.amazonaws.com/docs/Second_Life  _Case_Childrens_Memorial_EN.pdf | 2010 | Preparing for a Disaster Without Disrupting Patient Care: The Children's Memorial Hospital Chicago in Second Life |
| Novack | Medical Teacher | 2002 | A pilot test of WebOSCE: a system for assessing trainees' clinical skills via teleconference |
| Ohta | Journal of Telemedicine and Telecare | 2006 | Remote support for emergency medicine using a remote-control laser pointer |
| Okrainec | Surgical Endoscopy | 2013 | Feasibility of remote administration of the Fundamentals of Laparoscopic Surgery (FLS) skills test |
| Okrainec | Surgical Endoscopy | 2009 | Telesimulation: an effective method for teaching the fundamentals of laparoscopic surgery in resource-restricted countries |
| Packard | Journal of Nursing Education | 2019 | A Synchronous Interprofessional Patient Safety Simulation Integrating Distance Health Professions Students |
| Parsons | Canadian Journal of Emergency Medicine | 2017 | Iterative prototype development of a mobile tele-simulation unit for remote training: an update |
| Peisachovich | Cureus | 2020 | Implementing Virtual Simulated Person Methodology to Support the Shift to Online Learning: Technical Report |
| Pennington | American Journal of Respirology and Critical Care Medicine | 2018 | Evaluation of TEAM dynamics before and after remote simulation training utilizing CERTAIN platform |
| Posey | Journal of Nursing Regulation | 2018 | Comparing Nurse Practitioner Student Diagnostic Reasoning Outcomes in Telehealth and Face-to-Face Standardized Patient Encounters |
| Power | Journal of Paramedic Practice | 2011 | Enhancing the student learning experience through interactive virtual reality simulation |
| Prettyman | Journal of Nurse Practioners | 2018 | Objective Structured Clinical Examination From Virtually Anywhere! |
| Quinlin | Journal of the American Association of Nurse Practioners | 2020 | Development and implementation of an e-visit objective structured clinical examination to evaluate student ability to provide care by telehealth |
| Renouf | Cureus | 2017 | Emergency Management of Tension Pneumothorax for Health Professionals on Remote Cat Island Bahamas |
| Robinson-Reilly | Nurse Practioner | 2020 | Adding telehealth simulation into NP programs |
| Rogers | British Journal of Educational Technology | 2011 | Developing simulations in multi-user virtual environments to enhance healthcare education |
| Rosser | Surgical Technology International | 2017 | Design and Development of a Novel Distance Learning Telementoring System Using Off-the-Shelf Materials and Software. |
| Rossi | T+D | 2006 | Can Sims Save Your Life? |
| Rudolph | Nursing Education | 2017 | Integrating Telepresence Robots Into Nursing Simulation |
| Sa-Couto | MedEd Publish | 2020 | How to use telesimulation to reduce COVID-19 training challenges: A recipe with free online tools and a bit of imagination |
| Sampsel | Telemed J E Health | 2014 | Utility and Effectiveness of a Remote Telepresence Robotic System in Nursing Education in a Simulated Care Environment |
| Seibert | Nursing Education Perspectives | 2004 | Improving learning outcomes: integration of Standardized patients and Telemedicine Technology |
| Shao | Mayo Clin Proc Innov Qual Outcomes | 2018 | Feasibility of an International Remote Simulation Training Program in Critical Care Delivery: A Pilot Study |
| Shenai | Clinical Anatomy | 2011 | Virtual Interactive Presence and Augmented Reality (VIPAR) for Remote Surgical Assistance |
| Smith | Journal of Nursing Education | 2018 | Using Simulation to Teach Telehealth Nursing Competencies |
| Spence | Canadian Journal of Critical Care Nursing | 2018 | Dummies on the Go: Utilizing Telehealth During Pediatric Outreach Education |
| Stewart | Australasian Journal of Educational Technology | 2012 | On the MUVE or in Decline: Reflecting on the Sustainability of the Virtual Birth Centre Developed in Second Life |
| Stytz | Studies in Health Technology and Informatics | 1997 | A Distributed Virtual Environment Prototype  for Emergency Medical Procedures Training |
| Sudhir | MedEd Publish | 2020 | Adapting to the need of the hour: Communication skills simulation session using an online platform during COVID-19 |
| Suzuki | International Congress Series | 2004 | Tele-training simulation for the surgical robot system "da Vinci" |
| Sweeney Haney | International Journal Nursing Education Scholarship | 2018 | How to Prepare Interprofessional Teams in Two Weeks: An Innovative Education Program Nested in Telehealth |
| Taekman | Frontiers in Public Health | 2017 | A Novel Multiplayer Screen-Based Simulation Experience for African Learners Improved Confidence in Management of Postpartum Hemorrhage |
| Tagawa | Studies in Health Technology and Informatics | 2016 | Evaluation of Network-Based Minimally Invasive VR Surgery Simulator |
| Taylor | Studies in Health Technology and Informatics | 2011 | Single and Multi-User Virtual Patient Design in the Virtual World |
| Tiffany | Intelligent Systems Reference Library | 2014 | Chapter 9: Facilitating Learning Through Virtual Reality Simulation: Welcome to Nightingale Isle |
| Tilton | Nursing Education Perspectives | 2015 | Non-Acute-Care Virtual Simulation: Preparing Students to Provide Chronic Illness Care. |
| Tooley | Journal of Telemedicine and Telecare | 1999 | MultiMedia remote interactive medical simulation |
| Torres | Medical Education | 2020 | Transition to online is possible: Solution for simulation-based teaching during the COVID-19 pandemic |
| Treloar | Mil Med | 2001 | On-Site and Distance Education of Emergency Medicine Personnel with a Human Patient Simulator |
| Tschannen | Journal of Nursing Education and Practice | 2012 | Use of virtual simulations for improving knowledge transfer among baccalaureate nursing students |
| Vance | Journal of Adolescent Health | 2019 | Using Standardized Patient Encounters To Build Interdisciplinary Pediatric Learners’ Gender-Affirming Clinical Self-Efficacy And Skills In Caring For Transgender Youth |
| Venail | International Journal of Audiology | 2018 | Evaluation of otoscopy simulation as a training tool for real-time remote otoscopy |
| Verkuyl | Clinical Simulation in Nursing | 2018 | Comparison of Debriefing Methods after a Virtual Simulation: An Experiment |
| von Lubitz | Studies in Health Technology and Informatics | 2003 | Transatlantic medical education: preliminary data on distance-based high-fidelity human patient simulation training. |
| von Lubitz | Studies in Health Technology and Informatics | 2004 | Bioterrorism: Development of large scale medical readiness using multipoint distance bases simulation training |
| Weiner | Studies in Health Technology and Informatics | 2010 | Using the Virtual Reality World of Second Life to Teach Nursing Faculty Simulation Management |
| Wiech | Medical Science Monitor | 2019 | Use of Selected Telemedicine Tools in Monitoring Quality of In-Hospital Cardiopulmonary Resuscitation: A Prospective Observational Pilot Simulation Study |
| Wood | J STEM Outreach | 2018 | Pandem-Sim: Development and Pilot Testing of a Live Simulation of Infectious Disease Outbreaks |
| Youngblood | Simulation in Healthcare | 2008 | Design, Development, and Evaluation of an Online Virtual Emergency Department for Training Trauma Teams |
